# A Retrospective Observational Study on Telemedicine in Prescribing Low-Dose Pills for Patients with Dysmenorrhea

**DOI:** 10.1089/tmr.2023.0063

**Published:** 2024-01-24

**Authors:** Yumi Inoo, Hiroshi Iida, Norihito Yoshioka, Hideki Koyama, Yusuke Saigusa, Kentaro Kurasawa, Masahiko Inamori

**Affiliations:** ^1^ Department of Medical Education, Yokohama City University School of Medicine, Yokohama, Japan.; ^2^ Medley, Inc., Tokyo, Japan.; ^3^ The Medical Corporations of Tsuzuki-kai Tsuzuki Ladies' Clinic, Yokohama, Japan.; ^4^ The Medical Corporations of Ikuju-kai Isogo You Ladies' Clinic, Yokohama, Japan.; ^5^ Department of Biostatistics, Yokohama City University School of Medicine, Yokohama, Japan.; ^6^ Department of Obstetrics and Gynecology, Yokohama City University School of Medicine, Yokohama, Japan.

**Keywords:** telemedicine, patients with dysmenorrhea, retrospective study, LEP treatment

## Abstract

**Introduction:**

In Japan, telemedicine has gradually expanded due to deregulation in response to the COVID-19 pandemic. However, its current status remains unclear, as it is primarily provided by general practitioners. Meanwhile, telemedicine has begun to be utilized for low-dose estrogen-progestin (LEP) prescriptions for dysmenorrhea.

**Methods:**

We conducted a retrospective analysis of medical record data from two gynecology clinics and performed an exploratory evaluation between a group that combined telemedicine and in-person visits during the initial 6 months of LEP treatment, and another group with only in-person visits.

**Results:**

After propensity score matching, 89 and 83 patients were eligible for the telemedicine and in-person groups, respectively, with 53 patients in both. The characteristics of both groups were similar after matching. There were no significant differences in the probability of abnormal uterine bleeding during the first 6 months of treatment (25% and 43% in each group; *p* = 0.064), side effects, or treatment efficacy between the two groups. The withdrawal rate at 6 months was significantly higher in the telemedicine group than in the in-person group (13% and 0%, *p* = 0.013). The average copayment for patients who covered 30% of the total cost was also significantly higher in the telemedicine group after 1 and 3 months of LEP prescription.

**Conclusion:**

The appropriate combination of telemedicine and in-person visits is currently employed in hospital visits, which does not differ significantly from in-person visits. Given the retrospective nature of this study and the limited number of cases, further investigation is necessary in the future.

## Introduction

In Japan, telemedicine was historically prohibited, except in remote or island areas. However, in 2015, the Ministry of Health, Labour and Welfare (MHLW) permitted the use of telemedicine in regular clinical care. Although medical reimbursement for telemedicine was added in 2018, conditions became more stringent, which reduced its practicality. Hence, telemedicine was not widely adopted.

In 2018, the MHLW established guidelines for telemedicine, which defined it as “medical treatment involving communicating the results of the diagnosis and prescribing medical treatment in real time between a doctor and a patient via video chat, which includes both visual and auditory information.”^1^ In April 2020, temporary notices were issued to address the COVID-19 pandemic, and restrictions on eligible diseases and duration of in-person care were relaxed. This led to the gradual widespread use of telemedicine. Although temporary measures, such as medical fees for telemedicine, ended in July 2023, the guidelines and medical fees were revised in January 2022, with regulations being permanently relaxed.

Recently, telemedicine has been utilized in obstetrics and gynecology for the prescription of low-dose pills to manage dysmenorrhea. Japan has approved two types of low-dose pills: oral contraceptives (OC), prescribed mainly for contraceptive purposes and self-funded, and low-dose estrogen-progestin (LEP), prescribed for dysmenorrhea treatment and covered by insurance.

When prescribing LEP, establishing a continuous relationship of trust between the gynecologist and the patient is essential, as withdrawal may be necessary due to pregnancy or severe side effects. Since patients often experience side effects in the first 3 months after starting medication, the OC/LEP guidelines recommend monthly visits.^2^ A favorable combination with telemedicine is expected to reduce patient anxiety and ensure the appropriate continuation of treatment.

However, some doctors believe that the use of telemedicine may be disadvantageous for patients. In addition, the actual state of telemedicine use is unclear, partly because telemedicine is mainly utilized by general practitioners.

This retrospective observational study compared existing practice data between a combination of telemedicine and in-person visits and in-person visits alone. This exploratory study aimed to examine the differences in the probability of abnormal uterine bleeding, the most common complication, during the utilization of telemedicine.

## Methods

### Study design

This retrospective study compared two groups: a combination of telemedicine and in-person visits (hereafter referred to as the “telemedicine group”) and an in-person visits-only group. This study obtained data from two facilities: the Medical Corporations of Ikuju-kai Isogo You Ladies' Clinic (Facility A) and Tsuzuki-kai Tsuzuki Ladies' Clinic (Facility B).

### Variables

The primary outcome assessed was the probability of experiencing abnormal uterine bleeding during the first 6 months of LEP treatment. Any occurrence of petechia or breakthrough bleeding (excluding withdrawal bleeding) was evaluated as “bleeding,” whereas its absence was evaluated as “no bleeding.” The probabilities of “bleeding” were compared between the two groups. Secondary outcomes included other side effect-related items, status of continued visits, patient cost burden, and treatment efficacy, among others.

Basic patient information, the presence or absence of an organic dysmenorrhea diagnosis, blood pressure, types of LEP selected, dosing method (cyclic or continuous), side effect-related information, visit continuation status (including whether it occurred through telemedicine or in-person), patient cost burden, and the presence of patient efficacy records in the medical record were obtained.

### Setting and participants

Both groups included patients aged 20 years and older who were prescribed LEP for dysmenorrhea under insurance. The telemedicine group included patients treated between February 1, 2020 and October 31, 2021, at Facility A, and between October 1, 2019 and October 31, 2021, at Facility B. The in-person group included patients treated between May 1, 2018 and January 31, 2020, at Facility A, and between September 1, 2017 and September 30, 2019, at Facility B.

**FIG. 1.**
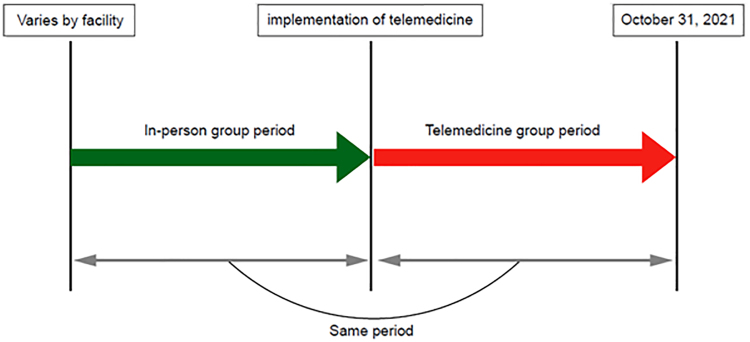
Timeline of data collection for telemedicine and in-person groups. Telemedicine group: from the implementation of telemedicine until October 31, 2021. In-person group: the same period as covered by the telemedicine group, retroactively from the time of implementation of telemedicine (the starting point varies depending on when telemedicine was implemented at each facility).

Exclusion criteria based on the 2020 OC/LEP guidelines for Japan^2^ were applied. Patients who received only in-person visits after the introduction of telemedicine at each facility were also excluded to reduce selection bias. Therefore, the periods covered by each group differed (Fig. 1). Data were extracted from the medical records of each facility through anonymized case registration forms for analysis.

For the target number of cases, the frequency of abnormal uterine bleeding during LEP varied in several studies, based on the type of medicine and study period.^3,4^ In a phase 3 Japanese study described in the LEP package insert, the mean and median incidence rates of all genital bleeding-related adverse events were 48.5% and 53.9%, respectively. Therefore, it was assumed that approximately 50% abnormal uterine bleeding would occur in each group. Considering the tolerance for some differences in the frequency of occurrence based on previous data, we set the margin of noninferiority at 20%.

In addition, a power at 80% and a significance level of 5% were selected, employing a chi-squared test with a case-count design. In total, 74 cases in each group were required for analysis after propensity score matching. Based on a propensity score matching rate of 70%, the required number of cases in each group was 106.

### Statistical methods

We used Fisher's exact test and the Mann–Whitney *U* test for categorical and continuous variables, respectively, to compare the differences between the telemedicine and in-person groups. All *p*-values were two-sided, and *p* < 0.05 was considered statistically significant.

For propensity score matching, the Caliper coefficient was set at 0.2 times the standard deviation of the log-transformed value of the propensity score. Furthermore, a 1:1 matching ratio was applied for the telemedicine and in-person groups. The calculation of the propensity score involved factors that may be related to abnormal uterine bleeding, including age, weight, smoking history, diagnosis of organic dysmenorrhea, blood pressure, type of LEP selected, dosing method, and history of LEP/OC administration. All statistical analyses were conducted using JMP PRO version 16.

### Ethical considerations

The requirement for informed consent was waived due to data anonymity. This study was approved by the Institutional Review Board of the Yokohama City University Life Science and Medical Research Ethics Committee for Humans and was conducted according to the principles of the Declaration of Helsinki.

### Telemedicine system and conflict of interest

Telemedicine at both facilities A and B used CLINICS^®^ (provided Medley, Inc.), a telemedicine system in Japan. However, this study did not aim to validate the telemedicine system. In addition, the co-researcher, the first author of this article, was both a visiting researcher at Yokohama City University and an employee of Medley, Inc. However, this study did not receive any funding from the company. The Conflict of Interest Review Committee granted permission to conduct this study on the condition that the aforementioned information was stated in the protocol, disclosure documents, and published article, and that the researcher did not engage in statistical analysis work.

## Results

### Patients' characteristics

A total of 89 and 83 eligible patients were categorized into the telemedicine and in-person groups, respectively. Propensity score matching resulted in 53 cases in both groups. Although the target number of cases was not reached, we analyzed the data obtained as part of this exploratory study. Pre-matched data exhibited some imbalance in smoking history, the presence of organic dysmenorrhea, types of LEP, and dosing method. However, the characteristics after propensity score matching were quite similar in both groups (Table 1).

**Table 1.** Pre- and Post-Matching Characteristics of Eligible Patients

	Before matching			After matching			Standard difference	Analysis methods
	Telemedicine group	In-person group	*p*	Telemedicine group	In-person group	*p*		
	*N* = 89	*N* = 83		*N* = 53	*N* = 53			
Age	29.21 ± 6.86	29.12 ± 7.81	0.69	28.08 ± 6.62	28.30 ± 7.41	0.94	−0.031	Mann–Whitney *U* test
Body weight (kg)	52.43 ± 6.33	54.04 ± 9.62	0.83	52.24 ± 6.47	53.67 ± 9.07	0.97	−0.18	Mann–Whitney *U* test
Height (cm)	159.4 ± 5.81	158.9 ± 4.05	0.27	159.1 ± 5.86	158.3 ± 3.67	0.48	0.15	Mann–Whitney *U* test
Smoking, *n* (%)								Fisher's exact test
Never	73 (82)	65 (78)	<0.001	50 (94)	51 (96)	1.00	0.089	
Past smoker	0 (0)	16 (19)		0 (0)	0 (0)		NA	
≥15 cigarettes per day	0 (0)	0 (0)		0 (0)	0 (0)		NA	
<15 cigarettes per day	0 (0)	0 (0)		0 (0)	0 (0)		NA	
Unknown	16 (19)	2 (2.4)		3 (0)	2 (3.8)		0.84	
Organic dysmenorrhea, *n* (%)								Fisher's exact test
None	60 (67)	37 (45)	0.002	35 (66)	28 (53)	0.32	0.015	
Any	19 (21)	39 (47)		13 (25)	20 (38)		0.29	
Unknown	10 (11)	7 (8.4)		5 (9.4)	5 (9.4)		NA	
Systolic blood pressure before LEP treatment (mmHg)	105.7 ± 11.2	103.4 ± 10.0	0.25	104.2 ± 11.8	104.0 ± 9.88	0.94	0.017	Mann–Whitney *U* test
Type of LEP, *n* (%)								Fisher's exact test
LUNABELL^®^ tablets LD	7 (7.9)	4 (4.8)	0.001	3 (5.7)	3 (5.7)	1.00	0	
LUNABELL^®^ tablets ULD	64 (72)	77 (93)		48 (91)	48 (91)		0	
YAZ^®^ combination tablets	12 (13)	1 (1.2)		1 (1.9)	1 (1.9)		0	
YAZ Flex^®^ combination tablets	6 (6.7)	1 (1.2)		1 (1.9)	1 (1.9)		0	
Dosing method, *n* (%)								Fisher's exact test
Cyclic dosing	77 (90)	82 (99)	0.032	52 (98)	52 (98)	1.00	0	
Extended dosing	6 (7.0)	1 (1.2)		1 (1.9)	1 (1.9)		0	
Both	3 (3.5)	0 (0)		0 (0)	0 (0)		0	
History of taking LEP and/or OC, *n* (%)								Fisher's exact test
Yes	24 (27)	12 (14)	0.06	8 (15)	8 (15)	1.00	0	
Just before	7 (7.9)	2 (2.4)		1 (1.9)	1 (1.9)		0	
Within 1 month	1 (1.1)	0 (0)		0 (0)	0 (0)		NA	
Within 2–3 months	2 (2.3)	0 (0)		0 (0)	0 (0)		0	
Within 4–6 months	1 (1.1)	1 (1.2)		0 (0)	0 (0)		NA	
Within 7–12 months	1 (1.1)	1 (1.2)		1 (1.9)	1 (1.9)		0	
>1 year ago	11 (12)	5 (6.0)		5 (9.4)	3 (5.7)		0.14	
Unknown	1 (1.1)	3 (3.6)		1 (1.9)	3 (5.7)		0.20	
No	65 (73)	71 (86)		45 (85)	45 (85)		0	

LEP, low-dose estrogen-progestin; OC, oral contraceptives.

The two groups did not differ significantly in the prevalence of comorbidities, at 21% and 23%, respectively (*p* = 0.88). The most common comorbidity in both groups was pollen allergy. Medical history was present in 21% and 0% in the telemedicine and in-person groups, respectively, indicating a significant difference (*p* < 0.001). However, the telemedicine group had no medical history that could have influenced abnormal uterine bleeding. The use of concomitant medications in the two groups was 15% and 17%, respectively (*p* = 0.31), with no significant differences or specific types of medications used (Table 2).

**Table 2.** Frequency and Description of Comorbidities, Medical History, and Concomitant Medication

	Telemedicine group	In-person group	*p*	Analysis methods
	*n* = 53	*n* = 53		
Presence of comorbidity				Fisher's exact test
Any	11 (21)	12 (23)	0.88	
Uterine fibroids (operated)	1 (1.9)	0 (0)		
Pollen allergy	4 (7.6)	5 (9.4)		
House dust allergy	1 (1.9)	0 (0)		
Allergic rhinitis	1 (1.9)	0 (0)		
Basedow's disease	1 (1.9)	0 (0)		
IgA nephropathy	1 (1.9)	0 (0)		
Migraine	2 (3.8)	0 (0)		
Diabetes	0 (0)	1 (1.9)		
Gastroesophageal reflux disease	0 (0)	1 (1.9)		
Anxiety disorder	0 (0)	2 (3.8)		
Insomnia	0 (0)	1 (1.9)		
None	40 (75)	38 (72)		
Unknown	2 (3.8)	3 (5.7)		
Presence of medical history				Fisher's exact test
Any	11 (21)	0 (0)	<0.001	
Appendicitis	3 (5.7)	0 (0)		
Inguinal hernia (operated)	1 (1.9)	0 (0)		
Canal of Nuck (operated)	1 (1.9)	0 (0)		
Gastric and duodenal ulcer	1 (1.9)	0 (0)		
Tonsillectomy	1 (1.9)	0 (0)		
Rubella	1 (1.9)	0 (0)		
Childhood asthma	1 (1.9)	0 (0)		
Viral hepatitis	1 (1.9)	0 (0)		
Infectious mononucleosis	1 (1.9)	0 (0)		
Ureteral stone	1 (1.9)	0 (0)		
Chronic sinusitis	1 (1.9)	0 (0)		
Ovarian endometrioma (operated)	1 (1.9)	0 (0)		
None	41 (77)	51 (96)		
Unknown	1 (1.9)	2 (3.8)		
Presence of concomitant medication				Fisher's exact test
Any	8 (15)	9 (17)	0.31	
Antihistamine	2 (3.8)	4 (7.6)		
Antithyroid drug	1 (1.9)	0 (0)		
Potassium iodide	1 (1.9)	0 (0)		
Proton pump inhibitor	1 (1.9)	0 (0)		
Iron preparations	1 (1.9)	0 (0)		
Vitamin preparations	1 (1.9)	2 (3.8)		
Antifibrinolytic agent	1 (1.9)	0 (0)		
Antibiotics	1 (1.9)	0 (0)		
Benzodiazepine derivative	1 (1.9)	3 (5.7)		
Aldosterone antagonist	0 (0)	1 (1.9)		
Sodium-glucose cotransporter 2	0 (0)	1 (1.9)		
Antifatty liver drug	0 (0)	1 (1.9)		
Selective serotonin reuptake inhibitor	0 (0)	1 (1.9)		
Antiepileptic drugs	0 (0)	1 (1.9)		
Herbal medicine	0 (0)	1 (1.9)		
None	41 (77)	35 (66)		
Unknown	4 (7.6)	9 (17)		

### Outcome data

#### Primary end-point

The probabilities of experiencing abnormal uterine bleeding during the first 6 months of treatment were 25% and 43% in the telemedicine and in-person groups, respectively. However, Fisher's exact test showed no significant difference between the two groups (*p* = 0.064) (Table 3).

**Table 3.** Rate of Experiencing Any Abnormal Uterine Bleeding Within 6 Months of Low-Dose Estrogen-Progestin Treatment

	Telemedicine group	In-person group	*p*
	*N* = 53	*N* = 53	
Abnormal uterine bleeding within 6 months after starting LEP, *n* (%)			Fisher's exact test
Yes	13 (25)	23 (43)	0.064
No	40 (75)	30 (57)	

#### Secondary end-points

##### Other side effects

The probability of occurrence of side effects recorded during the period under study was 38% in both groups. The probabilities of experiencing abnormal uterine bleeding from 6 months onward of LEP treatment were 7.6% and 11% in the telemedicine and in-person groups, respectively, which showed no significant differences. Nausea, headache, abdominal pain, and emotional instability were common symptoms, but no significant differences were observed in the probability of occurrence between the two groups (Supplementary Table S1).

##### Continuation of visits

The outpatient withdrawal rate at 6 months after the start of LEP treatment stood at 13% and 0% in the telemedicine and in-person groups, respectively, which was significantly higher in the telemedicine group (*p* = 0.013) (Table 4). Although a log-rank test was initially planned to compare LEP medication duration, it was considered unsuitable as the in-person treatment group started treatment earlier based on the time period. The graph provides treatment duration as a reference value (Supplementary Fig. S1).

**Table 4.** Visits at 6 Months After Starting Low-Dose Estrogen-Progestin Treatment

	Telemedicine group	In-person group	*p*
	*N* = 53	*N* = 53	
	Total	Total	
Visits at 6 months after starting LEP prescription, *n* (%)			Fisher's exact test
Yes	45 (85)	52 (98)	0.013
No	7 (13)	0 (0)	
Unknown	1 (1.9)	1 (1.9)	

The need for LEP included cases that did not continue treatment owing to a desire for pregnancy, age, and risk of complications. Nevertheless, the trend was generally similar in the duration of continued hospital visits after LEP initiation (Supplementary Fig. S2).

**FIG. 2.**
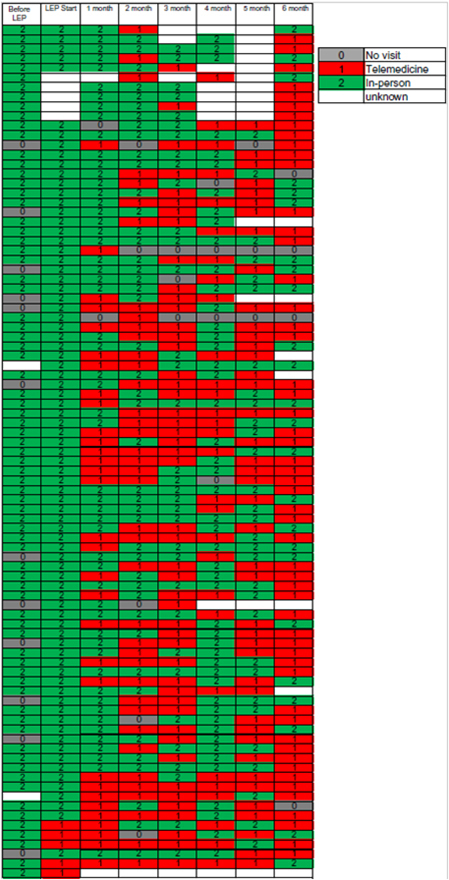
Telemedicine cases at 6 months after LEP prescription (in order of case registration). LEP, low-dose estrogen-progestin.

A review of the medical care format of the 89 patients in the telemedicine group before matching showed that almost all cases combined telemedicine and in-person care as appropriate (Fig. 2). The combinations varied from case to case.

##### Copayment

Since the most common copayment rate for both groups was 30%, only these groups were considered. After matching, the number of cases with 30% coverage were 48 (91%) and 51 (96%) in the telemedicine and in-person groups, respectively.

The data collected at the start of LEP prescription and after 1, 3, and 6 months of LEP administration were different. After 1 and 3 months, the average copayments were 3554.7 yen and 3513.0 yen in the telemedicine group, and 2695.7 yen and 3162.4 yen in the in-person group, respectively. The telemedicine group had significantly higher values (Table 5).

**Table 5.** Patient Cost Burden at Low-Dose Estrogen-Progestin Treatment Commencement and 1, 3, and 6 Months After Low-Dose Estrogen-Progestin Prescription (Yen)

	Telemedicine group			In-person group			*p*
	*N*	Mean (median)	Range	*N*	Mean (median)	Range	
LEP start	48	4792.1 (3280)	2518–6525	51	4232.8 (3170)	2480–5190	0.28
1 Month	47	3554.7 (2930)	2680–3580	49	2695.7 (2480)	2480–3217	<0.001
3 Months	44	3513.0 (2820)	2500–3430	49	3162.4 (2480)	2480–2840	0.022
6 Months	41	3286.8 (2680)	2500–3170	45	3114.5 (2630)	2480–3795	0.40

##### Efficacy of LEP treatment

In total, 17 (32%) and 23 (43%) patients confirmed treatment efficacy in the telemedicine and in-person groups, respectively, with no significant differences noted between the two groups (Table 6).

**Table 6.** Rate of Confirmed Low-Dose Estrogen-Progestin Effectiveness

	Telemedicine group	In-person group	*p*
	*N* = 53	*N* = 53	
	Total	Total	
Confirmation of effectiveness, *n* (%)			Fisher's exact test
Yes	17 (32)	23 (43)	0.080
No	32 (60)	30 (57)	
Unknown	4 (7.6)	0 (0)	

## Discussion

Telemedicine has become increasingly widespread in Japan, especially in clinics. Recent literature has emerged on telemedicine.^5,6^ However, many studies, especially clinical research, have been conducted in hospitals,^7,8^ and the actual status of telemedicine in clinics remains unclear.

Currently, telemedicine has limitations in the ability to obtain detailed physical information. This is because telemedicine is primarily based on medical interviews and visual examinations through video chats. As of 2023, there are no disease restrictions on telemedicine, but the range of suitable diseases for telemedicine is limited. Prescribing low-dose pills is a likely option. However, few studies have examined telemedicine and low-dose prescriptions.

Some studies have reported that approximately 1.6 million women in Japan suffer from functional dysmenorrhea.^9^ Furthermore, many of them do not receive appropriate treatment based on clinical guidelines in the early stages of the disease,^10^ leading to the exacerbation of their symptoms. Hence, early intervention by a gynecologist is considered beneficial. In this study, we evaluated the prescription practices in clinics providing telemedicine during insurance-covered treatment for dysmenorrhea.

The primary end-point, the probability of abnormal uterine bleeding during the first 6 months of LEP treatment, did not suggest that the use of telemedicine would result in different outcomes from in-person visits only. Although we did not determine whether complaints were made during telemedicine or in-person visits, it was still possible to identify the presence of abnormal uterine bleeding to a certain extent, even in the telemedicine group. Nevertheless, the in-person group tended to have a higher probability. It is possible that the sample size was smaller than the target, so the difference was not significant. Further study is needed, however, before we can conclude that in-person visits are more likely to pick up abnormal uterine bleeding.

Besides, the percentage of patients who visited the hospital 6 months after LEP initiation was significantly higher in the in-person group. We initially expected that telemedicine would make it easier for patients to continue their treatment; however, this study indicated the opposite result. Statistical evidence on the continuation rates of telemedicine is scarce. One systematic review reported an average dropout rate of 23% in 69 telemedicine studies, with a maximum of 83%.

However, it was difficult to establish a common definition of continuity rate, as it differed between studies.^11^ We analyzed the binominal value of whether the patient had visited; however, as some resumed visiting the clinic, this study could not conclude that telemedicine was more likely to cause patients to drop out. In fact, many patients in the telemedicine group continued to visit the clinic after 6 months (Supplementary Fig. S1). Whether telemedicine is more likely to encourage patients to continue treatment should be examined from a different perspective.

Regarding gynecological symptoms in the “Diseases/Conditions that can be treated continuously by telemedicine” outlined by The Japanese Medical Science Federation, dysmenorrhea was only listed as “functional dysmenorrhea for which organic disease is excluded and is treated only with painkillers.”^12^ Therefore, the consensus was that patients with dysmenorrhea and LEP should use telemedicine in combination with in-person treatments.

Since there were concerns that once patients used telemedicine, they would discontinue in-person consultations, we collected data on the frequency of telemedicine use. The Japanese guideline for telemedicine states in this basic principles that “an appropriate combination of in-person medical care is required.”^1^ This study suggests that the combination of telemedicine and in-person visits may vary, and that the choice of treatment is determined based on discussions between the physician and the patient. However, since the number of cases was limited and acceptable telemedicine continuity varied based on the medical condition, further studies are necessary.

Regarding cost-effectiveness, Arakawa et al. suggested that early physician consultation and guideline-based interventions were more cost-effective than self-care for patients with dysmenorrhea in Japan.^13^ Japan has a universal health insurance system, and although copayment rates vary according to age and annual income, the largest group of the population pays 30% of the cost. The amount reimbursed to medical institutions is set lower for telemedicine compared with in-person visits in Japan as of 2023.

Conversely, with the consent of the patient, uninsured expenses designated by the medical institution for system usage fee can be collected as “expenses for services not directly related to medical treatment benefits.” Thus, patients may have to pay higher copayments. In this study, patient copayments were compared between the two groups. There were no significant differences at the start of LEP treatment or after 6 months, however, higher in the telemedicine group at 1 and 3 months.

Although the uninsured copayments mentioned earlier may be a factor, these patient copayments may include the cost of treatment other than dysmenorrhea treatment. In addition, the reimbursement system during the study period and at the time of writing this article differed. Therefore, there is a possibility that it may change again due to systemic change and the result can only be used as a reference.

The symptom-relieving effects of LEP for dysmenorrhea have been demonstrated in Japan, and most were confirmed in approximately two to five LEP cycles.^14^ We initially believed that telemedicine might pose difficulties in documenting these effects; however, this was not the case in this study. Our study was retrospective and did not utilize quantitative measures, such as scores. Irrespective of whether the treatment effect was documented, there was no significant difference between the two groups.

The circumstance of telemedicine varies between countries due to differences in regulations and other factors. Although more studies are needed, evidence of the effectiveness of telemedicine and continuity of clinic visits is insufficient in Japan. This study alone will not be sufficient, but it may contribute to aiding a more comprehensive understanding of the topic.

### Limitations

The expected number of patients in this study was initially set at 212. However, the actual number of eligible patients meeting the criteria fell short of the initial expectations. Furthermore, due to circumstances within the facilities, it was not possible to examine a sufficient number of patients.

In addition, this study was retrospective in nature and conducted within localized facilities. Hence, the results may not be easily generalized to a wider population. Although propensity score matching was utilized to adjust for bias, cases that could not be matched were omitted, leading to the possibility of residual confounding. Furthermore, the primary end-point reflected the presence or absence of documented abnormal uterine bleeding and might not necessarily capture the actual probability of its occurrence. As this study was exploratory, and multiple end-points had missing values, it remained a secondary evaluation. Future large-scale prospective studies are warranted for more accurate results.

## Conclusion

This study compared the use of telemedicine and in-person treatment for patients with dysmenorrhea who were prescribed LEP within 6 months in a Japanese clinic. The study's primary end-point, the probability of abnormal uterine bleeding during the first 6 months of treatment, as well as side effects, clinic visits, and the financial burden on patients, were examined in an exploratory manner. The results suggested that the current approach, involving an appropriate combination of telemedicine and in-person care during clinic visits, does not differ significantly from relying solely on in-person care. Nevertheless, this study contributes to the understanding of the status of telemedicine in Japan. To address concerns in clinical practice, additional data on telemedicine for a range of diseases at various facilities need to be examined.

## Supplementary Material

Supplemental data

Supplemental data

Supplemental data

## Abbreviations Used

LEP: low-dose estrogen-progestin
MHLW: Ministry of Health, Labour and Welfare
OC: oral contraceptive
