# Supplementary material for: A Retrospective Observational Study on Telemedicine in Prescribing Low-Dose Pills for Patients with Dysmenorrhea
Source: Telemed Rep. 2024 Jan 24;5(1):2–11. doi: 10.1089/tmr.2023.0063 (PMC10927238; doi:10.1089/tmr.2023.0063)
Supplement: Supplemental data [file Suppl_TableS1.docx]

**Supplementary files**

Supplementary Table S1**.** Frequency and description of side effects
