# Supplementary material for: A Retrospective Observational Study on Telemedicine in Prescribing Low-Dose Pills for Patients with Dysmenorrhea
Source: Telemed Rep. 2024 Jan 24;5(1):2–11. doi: 10.1089/tmr.2023.0063 (PMC10927238; doi:10.1089/tmr.2023.0063)

Supplementary Figure S2**.** Duration of outpatient visits after starting LEP prescription at the time of data collection


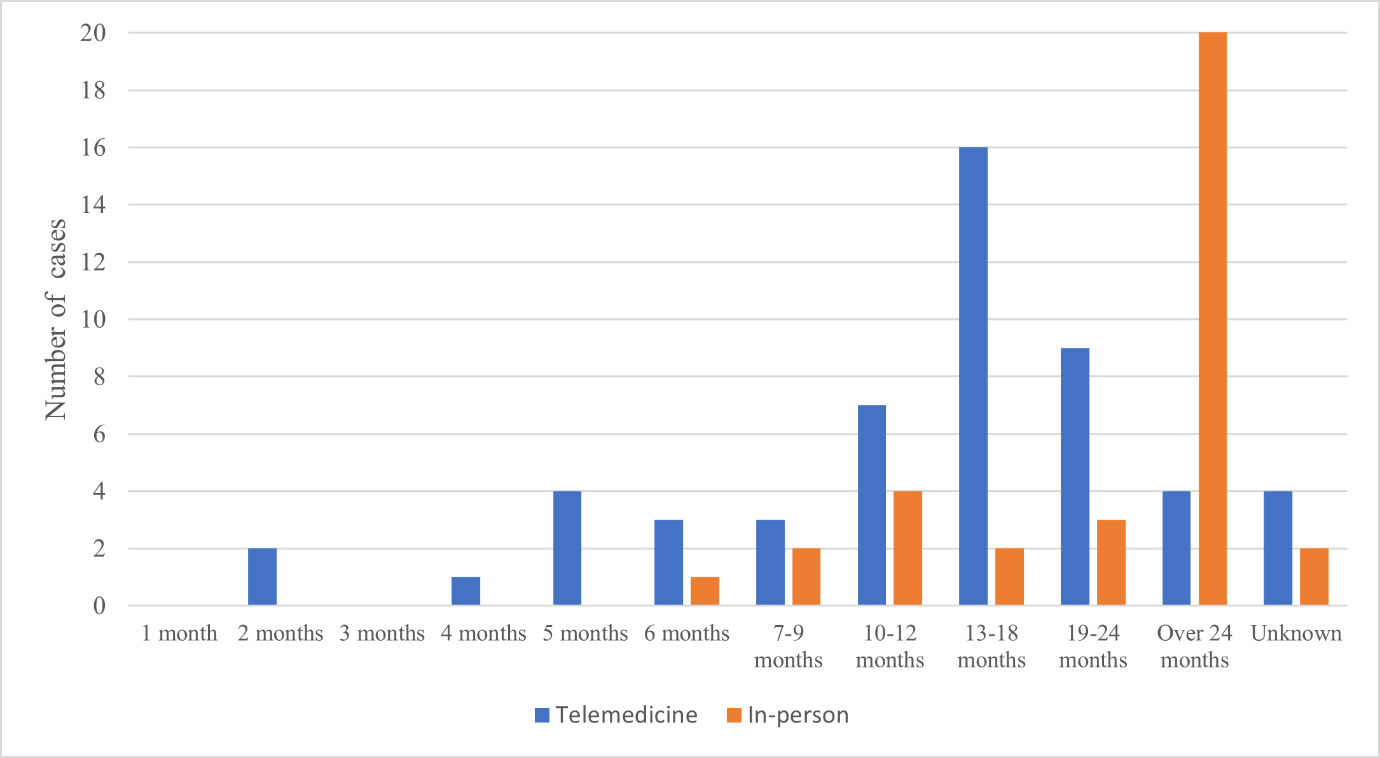

Supplement: Supplemental data [file Suppl_FigS2.docx]
